# Fournier’s Gangrene: Lessons Learned from Multimodal and Multidisciplinary Management of Perineal Necrotizing Fasciitis

**DOI:** 10.3389/fsurg.2017.00036

**Published:** 2017-07-10

**Authors:** Orestis Ioannidis, Loukiani Kitsikosta, Dimitris Tatsis, Ioannis Skandalos, Aggeliki Cheva, Aikaterini Gkioti, Ioannis Varnalidis, Savvas Symeonidis, Natalia Antigoni Savvala, Styliani Parpoudi, George K. Paraskevas, Manousos George Pramateftakis, Efstathios Kotidis, Ioannis Mantzoros, Konstantinos George Tsalis

**Affiliations:** ^1^Fourth Surgical Department, Medical School, Aristotle University of Thessaloniki, Thessaloniki, Greece; ^2^Department of Surgery, General Hospital “Agios Pavlos”, Thessaloniki, Greece; ^3^Department of Pathology, General Hospital “G. Papanikolaou”, Thessaloniki, Greece; ^4^Department of Microbiology, General Hospital “G. Papanikolaou”, Thessaloniki, Greece; ^5^Department of Plastic Surgery, General Hospital “G. Papanikolaou”, Thessaloniki, Greece; ^6^Department of Anatomy, Medical School, Aristotle University of Thessaloniki, Thessaloniki, Greece

**Keywords:** necrosis, perianal, perineum, polymicrobial, scrotum, surgical debridement

## Abstract

**Background:**

Fournier’s gangrene (FG) is a rapidly evolving necrotizing fasciitis of the perineum and the genital area, the scrotum as it most commonly affects man in the vast majority of cases. It is polymicrobial in origin, due to the synergistic action of anaerobes and aerobes and has a very high mortality. There are many predisposing factors including diabetes mellitus, alcoholism, immunosuppression, renal, and hepatic disease. The prognosis of the disease depends on a lot of factors including but not limited to patient age, disease extent, and comorbidities. The purpose of the study is to describe the experience of a general surgery department in the management of FG, to present the multimodal and multidisciplinary treatment of the disease, to identify predictors of mortality, and to make general surgeons familiar with the disease.

**Methods:**

The current retrospective study is presenting the experience of our general surgery department in the management of FG during the last 20 years. The clinical presentation and demographics of the patients were recorded. Also we recorded the laboratory data, the comorbidities, the etiology, and microbiology and the therapeutic interventions performed, and we calculated the various severity indexes. Patients were divided to survivors and non-survivors, and all the collected data were statistically analyzed to assess mortality factors using univariate and then multivariate analysis.

**Results:**

In our series, we treated a total of 24 patients with a mean age 58.9 years including 20 males (83.4%) and 4 females (16.6%). In most patients, a delay between disease onset and seeking of medical help was noted. Comorbidities were present in almost all patients (87.5%). All patients were submitted to extensive surgical debridements and received broad-spectrum antibiotics until microbiological culture results were received. Regarding all the collected data, there was no statistically significant difference between survivors and non-survivors except the presence of malignancy in non-survivors (*p* = 0.036) and the lower hemoglobin (*p* < 0.001) and hematocrit (*p* = 0.002) in non-survivors. However, multivariate analysis did not reveal any predictor of mortality.

**Conclusion:**

Early diagnosis, aggressive thorough surgical treatment, and administration of the proper antibiotic treatment comprise the cornerstone for the outcome of this disease. In small populations like in the present study, it is difficult to recognize any predictors of mortality and even the severity indexes, which take into account a lot of data cannot predict mortality.

## Introduction

Fournier’s gangrene (FG) is a surgical and urological emergency as it is a life threatening, potentially lethal, polymicrobial necrotizing fasciitis of the perineal and genital region affecting mainly males, but it can also present in females too (1–5). While this condition was known and has been described in sporadic case reports by the late eighteenth century (4, 6–17) it was only by the end of the nineteenth century that Jean Alfred Fournier provided a case series with a detailed description of the disease (13). However, until today, despite the fact, that in many circumstances the disease is treated by general surgeons and not by urologists, the description and analysis of FG is missing in most of the surgical textbooks. The purpose of the current retrospective study is to describe the experience of a general surgery department in the management of FG, to present the multimodal and multidisciplinary treatment of the disease, to identify, if possible predictors of mortality, including demographic, clinical, laboratory, etiological, microbiological, and surgical factors and also comorbidities and comorbidity and disease severity indexes, and finally to make general surgeons familiar with the disease.

## Materials and Methods

### Study Design

For the current retrospective study, the surgical and hospitalization records of our department from 1st January 1997 to 31st December 2016 were reviewed, searching for patients who have been diagnosed with FG or perineal gangrene or necrotizing fasciitis of the perineum. Ethical approval was not required for this study in accordance with the national and institutional guidelines. The diagnosis was based either on clinical examination, which revealed perineal skin necrosis or crepitus of the perineum, and/or on imaging studies revealing the presence of air in the perineal area. The final diagnosis of FG was established in all cases during surgery when the gray black, foul smelling gangrenous tissue was revealed.

The data recorded included demographics, clinical findings, medical history and comorbidities, laboratory findings, etiology and extent of the disease, therapeutic interventions, disease severity indexes, morbidity, mortality, and hospitalization. The outcome parameters of the study were in hospital mortality (defined as death from any cause during hospitalization), 30- and 90-day mortality (defined as death from any cause during the first 30 and 90 days after the procedure, respectively).

### Demographic Findings

The demographics data that were recorded regarding each patient included gender and age.

### Clinical Findings

The recorded clinical findings were as follows: symptoms, duration of symptoms prior to seeking medical treatment in the emergency department (ED), physical examination findings, vital signs {blood pressure (systolic, diastolic, and mean), heart rate [beats per minutes (bpm)], respiratory rate [breaths per minute (brpm)], and temperature}, presence of sepsis, severe sepsis, or septic shock. {Sepsis was defined as systemic inflammatory response syndrome (SIRS) in combination with a microbiologically or clinically documented infection. SIRS was defined as presence of at least two of the following criteria: temperature >38°C or <36°C, white blood cells (WBCs) >12,000/mm^3^, <4,000/mm^3^ or >10% of immature forms heart rate >90 bpm, respiratory rate >20 bpm or PaCO_2_ <32 mmHg. Severe sepsis was defined as sepsis along with tissue hypoperfusion (lactic acidosis with lactate >3 mmol/L) or hypotension (systolic blood pressure <90 or mean blood pressure <70 mmHg or a drop in systolic pressure ≥40 mmHg from normal) or organ dysfunction (18). Organ dysfunction variables were as follows: liver dysfunction [total bilirubin >2 mg/dL, activated partial thromboplastin time (aPPT) >60 s, or international normalized ratio (INR) >1.5], pulmonary dysfunction (arterial hypoxemia with PaO_2_/FiO_2_ <300), and renal dysfunction (diuresis <0.5 mL/kg/h for at least 2 h, creatinine >2 mg/dL, or an increase of >0.5 mg/dL), hematologic dysfunction (platelets <100,000/mm^3^), neurological dysfunction (disturbance of consciousness), cardiovascular dysfunction (hypotension in need of dopamine ≥5 µg/kg per minute or norepinephrine at any dose) (18, 19). Septic shock was defined as the presence of severe sepsis with hypotension despite adequate fluid resuscitation.}

### Medical History and Comorbidities

Past medical and surgical history was recorded, and the comorbidities considered were as follow: pulmonary disease, renal disease, heart disease, liver disease, diabetes mellitus, hypertension, hyperlipidemia, malignancy, peripheral vascular disease, psychiatric disease, obesity, and immunosuppression. Also smoking and alcoholism were recorded. Pulmonary disease was defined as either emphysema or chronic obstructive pulmonary disease. Renal disease was defined as serum creatinine >1.7 mg/dL (20). Heart disease was considered as presence of any of the following: angina pectoris, past myocardial infraction, coronary disease, coronary artery bypass graft surgery, signs of myocardial ischemia on the electrocardiogram, valvular heart disease, heart failure, and atrial fibrillation (20). Liver disease was defined as the presence of any of the following: chronic hepatitis B or C, liver cirrhosis (21). Diabetes mellitus was considered present if the patient had a fasting blood glucose >126 mg/dL or was on antidiabetic treatment with diet, oral drugs or insulin. Hypertension was defined as SP >150 mmHg or DP >90 mmHg or if the patient was receiving antihypertensive treatment (20). Hyperlipidemia was defined as total cholesterol levels ≥200 mg/dL, LDL levels ≥100 mg/dL, or the patient was under medical treatment. Malignancy was considered present if the patient had any active solid or hematologic malignancy. Peripheral vascular disease was considered present if the patient had a history rest pain, of claudication, or ischemic gangrene, or if he had undergone a previous intervention surgical or percutaneous (22), or as an ankle brachial index of less than 0.9. Psychiatric disease was considered present if the patient had depression, bipolar disorder, schizophrenia or if he was receiving drugs (23, 24). Obesity was defined as body mass index (BMI) >30. Immunosuppression was defined as leukopenia, chronic immunosuppressive treatment or any active solid or hematologic malignancy (25). Patients were defined as smokers if they had been smoking at least 10 cigarettes per day, until admission or if there was a history of at least 20 years of nicotine use given up not more than 10 years ago. Alcoholism was defined as the presence of either alcohol dependence or alcohol abuse. Also the Charlson comorbidity index (CCI) and the Age Adjusted Charlson comorbidity index (AACCI) were calculated.

### Laboratory Findings

The laboratory tests studied were hemoglobin, hematocrit, platelet count (PLT), WBC, glucose, serum creatinine, serum urea, serum total protein, serum albumin, serum globulin, liver function tests [including total bilirubin, gamma-glutamyl transferase, alkaline phosphatase, alanine transaminase (ALT-SGPT), and aspartate transaminase (AST-SGOT)], sodium (Na^+^), potassium (K^+^), calcium (Ca), lactate dehydrogenase, creatine kinase, coagulation tests [prothrombin time, international normalized ratio (INR), and aPPT], cholesterol levels, C-reactive protein (CRP), and gas analysis (including PaCO_2_, PaO_2_, bicarbonate, lactic acid, and pH).

### Etiology and Extent

Furthermore, the etiology of FG and microbiological test results (Gram positive, Gram negative, anaerobic) were recorded. The extent of FG indicating the affected body surface was assessed using modified body surface area nomograms used routinely to calculate the extent of burn injuries (26) according to which the perineum, scrotum, and penis account for 1% each and each ischiorectal fossa for 2.5% (18).

### Therapeutic Interventions

Antibiotics regimen administrated, the time interval from hospital admission to surgical treatment, the type and extent of surgery, need for colostomy and type of colostomy, need for urostomy, the use of a rectal diversion system, duration of surgery, number of surgical debridements performed, perioperative transfusion and use of vasoactive drugs was also recorded. Also, the use of hyperbaric oxygen and vacuum-assisted closure (VAC) were recorded. We also recorded the type of wound healing either as healing by secondary intention or by reconstruction with a skin graft performed by a plastic surgeon.

### Disease Severity Indexes

We calculated also the acute physiology and chronic health evaluation II severity score (APACHE II), the Fournier’s Gangrene Severity Index (FGSI) score, which has been created by Laor and colleagues in 1995 by modifying the APACHE II score (26). The nine parameters studied are heart rate, respiratory rate, temperature, hematocrit, leukocyte count, serum sodium, potassium, creatinine, and bicarbonate levels, and the deviation from normal is graded in a scale from 0 to 4 to a maximum of 36 points. The values are added to calculate the FGSI score (26). Moreover, we also calculated the Uludag FGSI that also takes into account age and dissemination to a maximum of 43 points (18). Finally, we calculated the laboratory risk indicator for necrotizing fasciitis (LRINEC) which is based on the values of CRP, WBC, hemoglobin, serum sodium, serum creatinine and plasma glucose (11).

### Morbidity, Mortality, and Hospitalization

In addition, complications and morbidity, the intensive care unit (ICU) stay, total hospitalization time, hospitalization in the surgical department, and hospitalization in plastic surgery department, and mortality were recorded.

### Statistical Analysis

Statistical analysis was performed using the Statistical Package for Social Sciences for windows version 20.0. The measured quantitative values were checked for normality. Values with normal distribution were expressed as mean ± SD and compared with parametric independent samples *t*-test, while values without normal distribution were expressed as median and interquartile range and compared to a non-parametric Mann–Whitney test. The measured qualitative values were expressed as frequency and percentage and compared with the chi-square test and Fisher’s exact test. Values found significant in the univariate analysis were used for multivariate analysis with a binary logistic regression model. The difference was considered statistically significant at *p* < 0.05 as the confidence interval was 95%.

## Results

**Figure 1:**
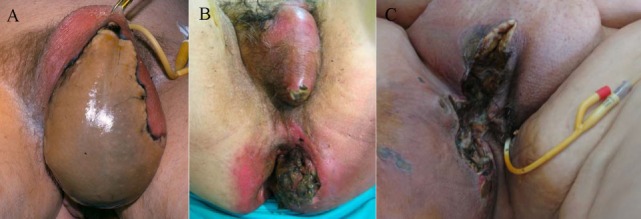
Skin necrosis of the scrotum **(A)**, scrotum, perineal and perianal region **(B)** in males and of the genital, and perineal region **(C)** in a female.

During the last 20 years a total of 24 patients were diagnosed and treated in our surgical department with the diagnosis of FG. The mean age was 58.9 ± 11.3 years (45–79 years), while 20 (83.3%) patients were males and 4 (16.7%) were females. The median time between disease onset to hospital admission was 2 days. Clinically the majority of patients (21 patients—87.5%) presented with local signs of inflammation in the perineal area including pain, heat, erythema, and local edema. The presence of local abscess was evident in eight patients (33.3%). Skin necrosis was present in 13 patients (54.2%), 3 (12.5%) of whom had a foul smelling local discharge, while crepitation was also present in 5 patients (20.8%). The disease involved the scrotum in males and the genital region in females in 19 cases (79.2%), the perineum in 22 cases (91.7%) and the perianal region in 13 cases (54.2%) (Figure 1). Most patients (18 patients—75%) were febrile, tachycardic with increased respiratory rate fulfilling the criteria of SIRS and sepsis (all patients were considered to have a clinically documented infection), nine patients (37.5%) presented with severe sepsis, and three patients (12.5%) with septic shock. There was no statistically significant difference between survivors and non-survivors regarding the demographics and clinical presentation of the disease (Table 1).

**Table 1:** Patient demographics and clinical presentation.

Variable	Total	Non-survivors	Survivors	*p*
Age (years)	58.9 ± 11.3	57.4 ± 10.9	58.8 ± 11.9	0.749
Male/female	20/4	4/1 (80%/20%)	16/3	0.822
HR (bpm)	93.1 ± 16.3	97 ± 18.2	93.3 ± 15.6	0.554
RR (brpm)	20.9 ± 5	23.6 ± 6.4	20 ± 4.6	0.168
SAP (mmHg)	103.1 ± 18.9	98 ± 19.2	104.3 ± 19.7	0.503
DAP (mmHg)	62.1 ± 11.7	60.3 ± 12	66 ± 11.4	0.412
MAP (mmHg)	78.8 ± 11.1	77 ± 11.9	82 ± 7.6	0.474
Temperature (°C)	37.9 ± 1.1	38.2 ± 1.3	37.9 ± 1.1	0.563
Diuresis (cc)	1,586 ± 593	1,400 ± 612	1,660 ± 611	0.436
Time from symptoms to emergency department (days)	2 (1)	3 (1.5)	2 (1)	0.410
Body surface (%)	3 (2)	3 (1.5)	3 (3)	0.528
Sepsis	18 (75%)	4 (80%)	14 (73.7%)	0.772
Severe sepsis	9 (37.5%)	2 (40%)	7 (36.8%)	0.897
Septic shock	3 (12.5%)	1 (20%)	2 (10.5%)	0.521

Neutrophilic leukocytosis was evident in the majority of patients (22 of 24—91.6%) while inflammation indicators (CRP) were increased in 23 patients (95.8%). The other laboratory test results did not differ significantly among survivors and non-survivors except from hematocrit and hemoglobin which were statistically significant lower in non-survivors (Table 2).

**Table 2:** Laboratory tests.

Variable	Total	Non-survivors	Survivors	*p*
Hemoglobin (g/dL)	11.5 ± 1.4	9.8 ± 0.9	12 ± 1.1	<0.001
Hematocrit (%)	33.9 ± 4.8	28.5 ± 3.8	35.3 ± 3.7	0.002
PLT (K/μL)	226,000 (222,500)	149,000 (294,500)	226,000 (229,000)	0.506
White blood cell (total/mm^3^)	15,000 (8,360)	17,600 (16,860)	14,700 (9,700)	0.619
Glucose (mg/dL)	100 (115)	120 (105)	100 (120)	0.618
Creatinine (mg/dL)	0.75 (0.6)	1.29 (1.2)	0.75 (0.61)	0.135
Urea (mg/dL)	33 (13.5)	40 (71.5)	32 (14)	0.198
Total protein (g/dL)	4.85 ± 0.49	4.86 ± 0.58	4.8 ± 0.47	0.965
Albumin (g/dL)	2.44 ± 0.29	2.41 ± 0.27	2.44 ± 0.31	0.777
Globulin (g/dL)	2.63 ± 0.32	2.65 ± 0.36	2.62 ± 0.33	0.910
Bilirubin (mg/dL)	0.84 (0.62)	0.88 (8.16)	0.84 (0.6)	0.934
Gamma-glutamyl transferase (U/L)	50 (37)	50 (42)	30 (38)	0.9
Alkaline phosphatase (U/L)	99 (50)	95 (401)	99 (50)	0.77
ALT (U/L)	24 (13)	20 (9.5)	26 (15)	0.868
AST (IU/L)	26 (14)	23 (70)	29 (12)	0.934
Na^+^ (mmol/L)	136 (7)	131 (7)	138 (6)	0.183
K^+^ (mmol/L)	3.9 (0.3)	4 (0.72)	3.9 (0.3)	0.707
Ca (mg/dL)	9.2 (1.3)	9.1 (0.9)	9.6 (1.7)	0.465
Lactate dehydrogenase (U/L)	169 (140)	138 (137.5)	177 (136)	0.77
Creatine kinase (U/L)	64 (62)	50 (118)	64 (46)	0.967
Prothrombin time (s)	14.8 ± 2.9	17.1 ± 4.9	14.2 ± 2.5	0.251
Activated partial thromboplastin time (s)	32 (2)	32 (0.95)	32 (3)	0.523
International normalized ratio	1.25 ± 0.24	1.43 ± 0.4	1.2 ± 0.13	0.255
Cholesterol (mg/dL)	210 (70)	210 (65)	225 (60)	0.803
C-reactive protein (mg/dL)	3.2 (1.9)	3.0 (2.1)	3.2 (1.6)	0.298
PaCO_2_ (mmHg)	32.5 ± 5.8	29.4 ± 5.1	32.9 ± 5.5	0.182
PaO_2_ (mmHg)	97.2 (19)	92.1 (7.3)	100 (18.9)	0.143
Bicarbonate (mmol/L)	21.2 ± 1.9	20.8 ± 1.3	21.5 ± 2	0.595
Lactic acid (mmol/L)	1.36 ± 0.46	1.36 ± 0.53	1.35 ± 0.46	0.992
pH	7.36 ± 0.09	7.35 ± 0.15	7.37 ± 0.08	0.717

Regarding comorbidities, three patients (12.5%) had no comorbidities, while seven patients (29.1%) presented with diabetes mellitus, of whom one patient (4.2%) was diagnosed during hospitalization. Also, seven patients had hyperlipidemia (29.1%), and six patients had peripheral vascular disease (25%). Hypertension was evident in six patients (25%), heart disease in five patients (20.8%), obesity in five patients (20.8%), pulmonary disease in four patients (16.6%), and immunosuppression in four patients (16.7%). Two patients (8.3%) presented with renal failure, two patients with liver disease (8.3%), two patients with malignancy (8.3%), and two patients with psychiatric disease (8.3%). Nine patients (37.5%) were smokers while five patients (20.8%) were alcoholics. The median BMI of the patients was 27. The median score in CCI was 2, and the median score in age-adjusted CCI was 3. With the exception of malignancy that was statistically significant more common in non-survivors than survivors (*p* = 0.036), there was no other statistically significant difference in comorbidities between non-survivors and survivors (Table 3). Regarding the history of surgical or urologic intervention, one patient had an inguinal hernia surgery, two patients had appendectomy, one patient had surgery of anal fissure, one patient had hemorrhoidectomy, one patient had been submitted to total mesorectal excision, and one patient had transurethral prostatectomy.

**Table 3:** Comorbidities.

Variable	Total (24)	Non-survivors (5)	Survivors (19)	*p*
Pulmonary disease	4 (16.6%)	2 (40%)	2 (10.5%)	0.179
Renal disease	2 (8.3%)	1 (20%)	1 (5.2%)	0.380
Heart disease	5 (20.8%)	1 (20%)	4 (21.0%)	0.959
Liver disease	2 (8.3%)	0(0%)	2 (10.5%)	0.449
Diabetes mellitus	7 (29.1%)	2 (40%)	5 (26.3%)	0.608
Hypertension	6 (25%)	1 (20%)	5 (26.3%)	0.772
Hyperlipidemia	7 (29.1%)	1 (20%)	6 (31.5%)	0.612
Malignancy	2 (8.3%)	2 (40%)	0 (0%)	0.036
Peripheral vascular disease	6 (25%)	1 (20%)	5 (26.3%)	0.772
Psychiatric disease	2 (8.3%)	1 (20%)	1 (5.2%)	0.380
Obesity	5 (20.8%)	1 (20%)	4 (21.0%)	0.959
Immunosuppression	4 (16.6%)	2 (40%)	2 (10.5%)	0.179
Smoking	9 (37.5%)	1 (20%)	8 (42.1%)	0.615
Alcoholism	5 (20.8%)	0 (0%)	5 (26.3%)	0.544
Charlson Comorbidity Index	2 (1.5)	3 (3.5)	2 (2)	0.084
Age Adjusted Charlson Comorbidity Index	3 (1)	3 (2)	3 (1)	0.156
Body mass index (kg/cm^2^)	27 (9.5)	29 (24.5)	26 (10)	0.482

**Figure 2:**
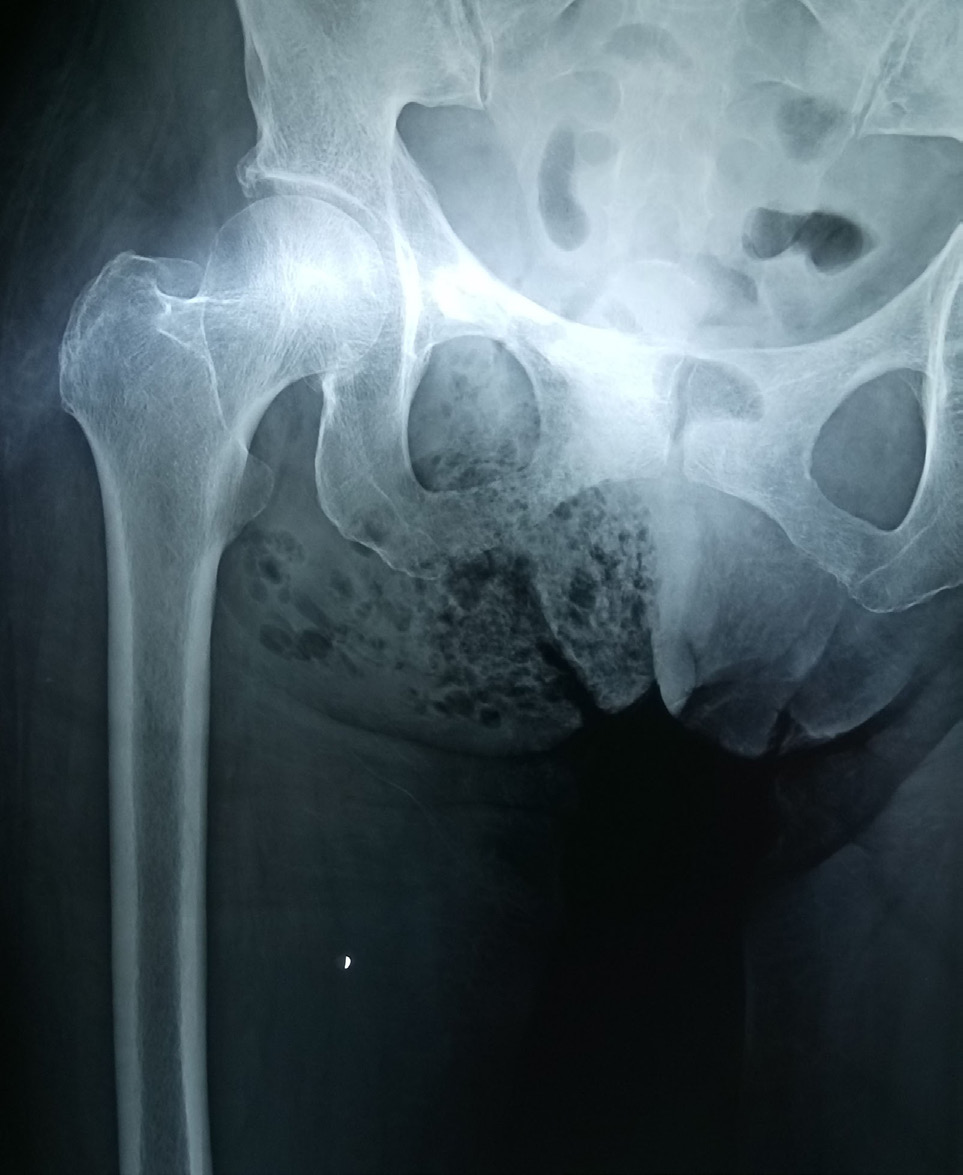
Pelvic X-ray showing the presence of air in the soft tissue.

Pelvic X-ray was performed in all patients and revealed the presence of air in soft tissues in 18 of them (75%) (Figure 2). Perineal and scrotum ultrasound was performed in one patient (4.2%) showing diffuse marked thickening of skin with multiple echogenic foci with associated dirty shadowing which was consistent with the presence of gas. Pelvic CT was performed in four patients (16.7%), and pelvic MRI in one patient (4.2%), who in fact presented to the ED with an MRI already performed previously, revealing extended local inflammation, soft tissue thickening, stranding of fat surrounding the involved structures and the presence of soft tissue air.

The initial infection origin in 10 patients (41.6%) was colorectal, of whom one patient had local recurrence of rectal cancer. Five patients (20.8%) presented with local skin infection or perineal abscess, while in four patients (16.7%) the initial port of entry was the urogenital tract. Three patients (12.5%) presented with local trauma in the area while two patients (8.3%) had idiopathic Fournier gangrene. The most common isolated bacteria were Gram negative and especially *Escherichia coli* in 11 patients (45.8%), followed in frequency by *Klebsiella pneumoniae* in three patients (12.5%), *Pseudomonas aeruginosa* in three patients (12.5%), *Acinetobacter baumannii* in two patients (8.3%), *Proteus mirabilis* in two patients (8.3%), and *Providencia stuartii* in one patient (4.2%). In same patients more than 1 g negative bacteria were present. Microbial cultures revealed also Gram positive bacteria and specifically *Staphylococcus aureus* in three patients (12.5%), *Staphylococcus epidermidis* in one patient (4.2%), *Streptococcus* species in two patients (8.3%), and *Enterococcus* species in two patients (8.3%). The anaerobe bacteria isolated from the microbial cultures were *Bacteroides* species in 11 patients (45.8%) and *Enterobacter* species in five patients (20.8%). Thirteen patients (54.1%) had a polymicrobial infection isolating more than one type of bacteria. There was statistically significant difference between survivors and non-survivors regarding the etiology and microbiology of the disease (Table 4).

**Table 4:** Etiology and microbiology.

Variable	Total (24)	Non-survivors (5)	Survivors (19)	*p*
Trauma	3 (12.5%)	1 (20%)	2 (10.5%)	0.521
Urogenital	4 (16.6%)	1 (20%)	3(15.7%)	0.822
Skin infection–abscess	5 (20.8%)	1 (20%)	4 (21.0%)	0.959
Idiopathic	2 (8.3%)	0 (0%)	2 (10.5%)	0.449
Colorectal	10 (41.6%)	2 (40%)	8 (42.1%)	0.668
Gram (−)	17 (70.8%)	4 (80%)	13 (68.4%)	0.612
Gram (+)	6 (25%)	1 (20%)	5 (26.3%)	0.772
Anaerobes	11 (45.8%)	2 (40%)	9 (47.4%)	0.769
Polymicrobial infection	13 (54.1%)	3(60%)	10 (52.6%)	0.769

The administered empirical antibiotic regimens were penicillin-type antibiotics (piperacillin with tazobactam) in 12 patients, third-generation cephalosporins (cefotaxime, ceftriaxone, ceftazidime) in 6 patients (25%), carbapenems (imipenem/cilastatin, meropenem) in 3 patients (12.5%), and quinolones (ciprofloxacin) in 3 patients (12.5%). In these antibiotic regimens, aminoglycosides (amikacin or gentamicin) were added in eight patients. Metronidazole was added in 20 patients (83.3%), clindamycin in 4 patients (16.7%), and antibiotics for Gram-positive bacteria (linezolid, daptomycin, and teicoplanin) were added in 4 patients (16.7%). Antibiotics regiments were changed according to culture susceptibility tests. The median duration of antibiotic administration was 16 days.

**Figure 3:**
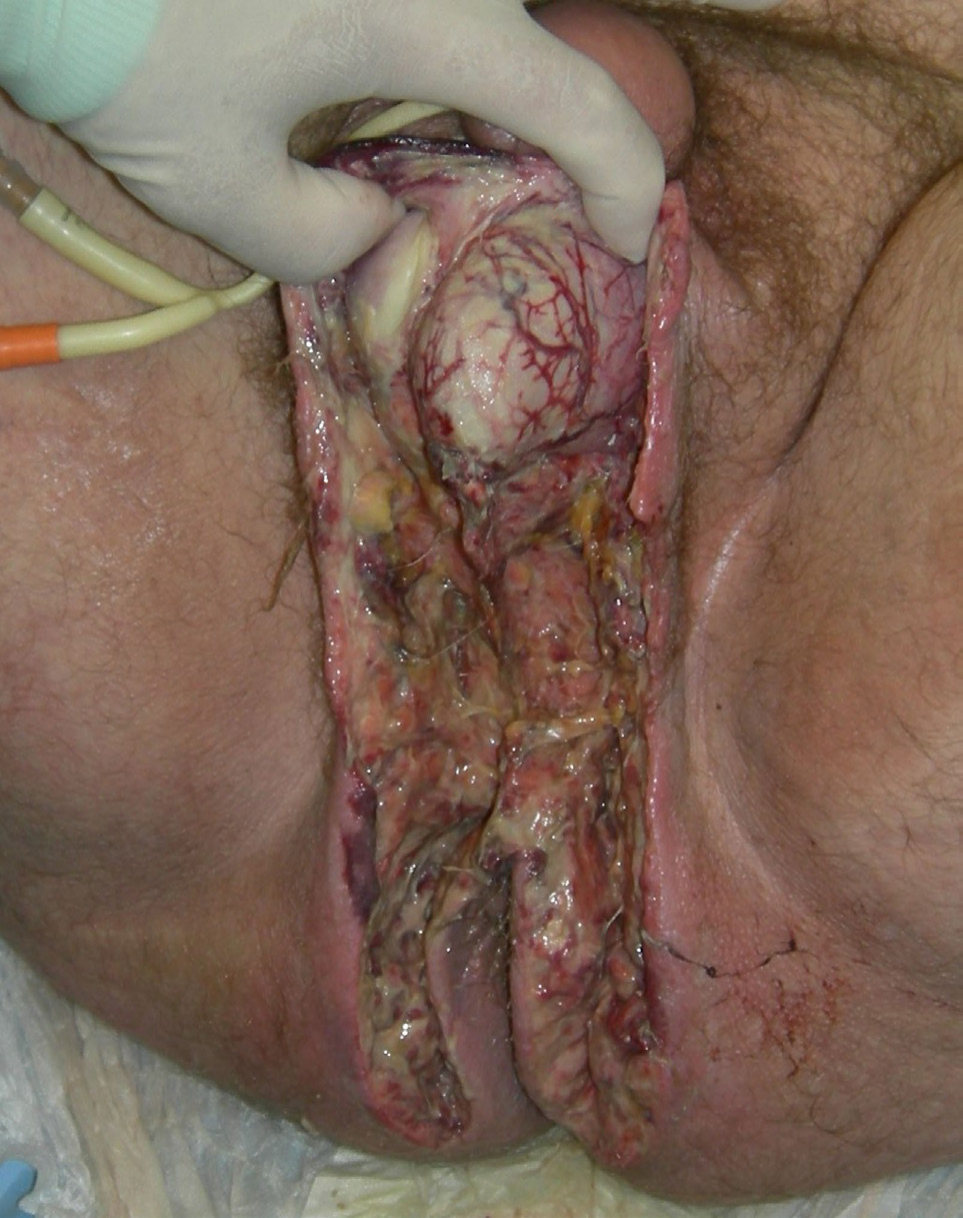
Extended surgical debridement of the scrotal, perineal, and perianal area.

**Figure 4:**
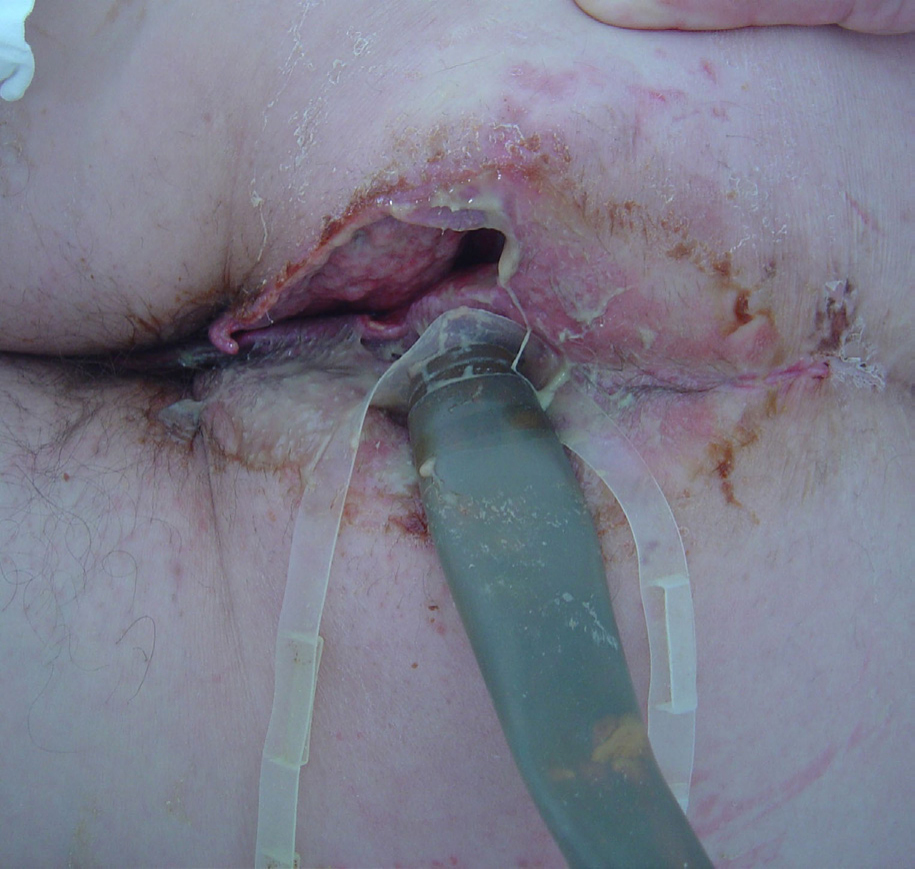
Rectal diversion device.

**Figure 5:**
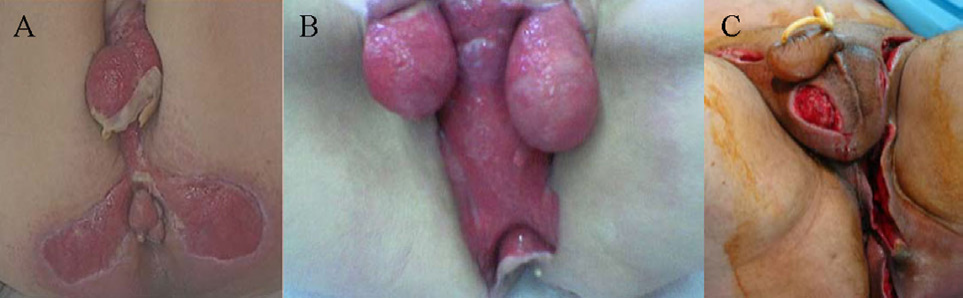
Granulomatosis of the surgical wounds following extended surgical debridement and daily dressing changes of the scrotum, perineal, and perianal region **(A)**, of the scrotum and perineum **(B)**, and of the scrotum and left inguinal region **(C)**.

The median time from admission to surgery was 4 h with the exception of one patient who has been admitted to the internal medicine department for unknown fever investigation, and the diagnosis was delayed for 48 h and the median surgical time was 55 min. Surgical debridement in all cases involved the perineal and scrotum area in males and the perineal and genital region in females, and in 11 patients (45.8%) involved the perianal area. The median Body surface affected by FG was 3%, and there was no statistically significant difference between non-survivors and survivors. Extensive and aggressive surgical debridement, with resection of necrotic or infected tissue and tissue of doubtful viability until viable tissue was encountered, was necessary in all patients (Figure 3). Four of the patients (16.7%) were submitted during the initial operation to loop colostomy, three of whom (12.5%) were submitted to sigmoidostomy and one patient (4.2%) to transverse colostomy. During operation, the median blood transfusion was 1 U of (red blood cells) RBC per patient, and seven patients needed vasoactive drugs. Rectal diversion device was used in two patients (8.3%) (Figure 4), while none of the patients needed urostomy. Thirteen patients were submitted to only one surgical debridement while seven patients (29.1%) were submitted to two surgical debridements and four patients (16.7%) had more than two surgical debridements. Following extended surgical debridement the surgical wounds were washed daily with plenty of hydrogen peroxide and povidone iodine, until the development of granulomatosis (Figure 5). Hyperbaric oxygen therapy was employed for three patients (12.5%), and VAC therapy in one patient (4.2%). There was statistically significant difference between survivors and non-survivors regarding the demographics and clinical presentation of the disease (Table 5).

**Table 5:** Intervention and hospitalization.

Variable	Total (24)	Non-survivors (5)	Survivors (19)	*p*
Time from emergency department to OR (h)	4 (2)	3 (2)	4 (2.75)	0.086
Surgical time (min)	55 (21.5)	60 (27.5)	55 (27)	0.647
Number of surgical debridements	1 (1)	2 (2)	1.5 (1)	0.631
Transfusion (RBC)	1 (2)	1 (2)	1 (2)	0.405
Vasoactive drugs	7 (29.1%)	2(40%)	5 (26.3%)	0.608
Colostomy	4(16.7%)	1	3	0.822
Rectal diversion	2(8.3%)	0	2	0.449
Urostomy	0 (0%)	0	0	1
Hyperbaric oxygen	3 (12.5%)	0	3	0.342
Vacuum-assisted closure	1	0	1	0.600
Total hospitalization (days)	27(38.5)	3 (16.5)	40 (39)	0.008
Intensive care unit stay (days)	0 (1.5)	0 (1.5)	0 (2)	0.961
Surgical department stay (days)	16 (34.5)	3 (18)	25 (34)	0.075

**Figure 6:**
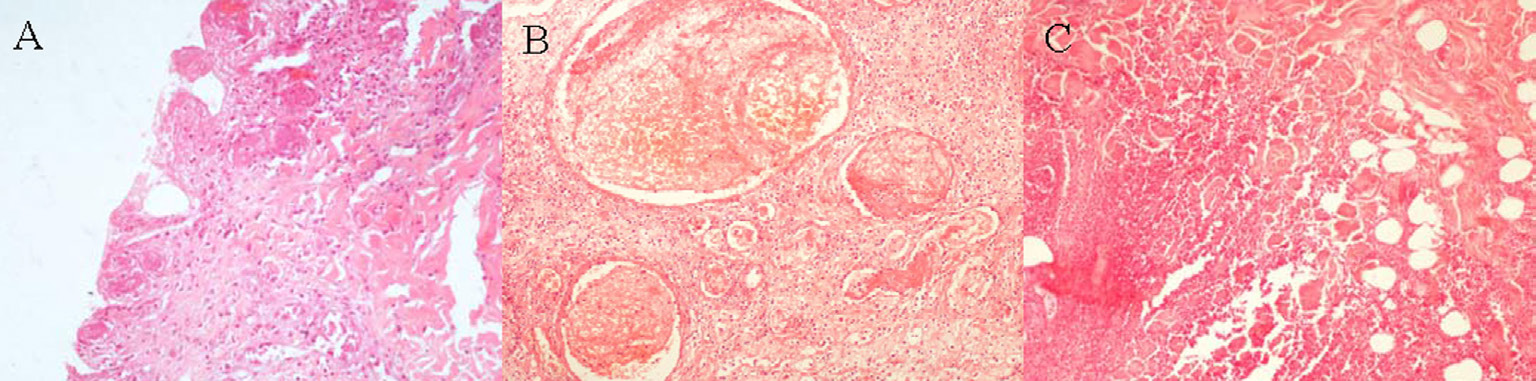
Histopathologic findings in Fournier’s gangrene revealing neutrophilic exudate and epidermis necrosis **(A)**, thrombosis of vessels **(B)**, and abscessation and necrosis of subcutaneous fat tissue **(C)** (hematoxylin and eosin staining ×200).

Histopathological analysis revealed ulceration of the epidermis, the presence of neutrophilic exudate, thrombosed vessels, and necrosis, and abscessation of the subcutaneous fat tissue (Figure 6).

The median prognostic severity index scores were 10 for APACHE II, 5 for FGSI, 6 for Uludag FGSI, and 5 for LRINEC and presented no statistically significant difference among survivors and non-survivors (Table 6).

**Table 6:** Severity index and prognostic scores.

Variable	Total	Non-survivors	Survivors	*p*
APACHE II	10 (3.5)	14 (12)	10 (2)	0.142
FGSI	5 (5)	5 (9)	2 (4)	0.179
Uludag FGSI	6 (9)	9 (6)	5 (3)	0.062
LRINEC	5 (4)	7 (4.5)	5 (4)	0.145

Regarding morbidity and complications, besides severe sepsis and septic shock, four patients were diagnosed with pneumonia, three patients developed multiple organ dysfunction syndrome (MODS), three patients with bacteremia, two patients with acute renal failure, one patient with pulmonary embolism, one patient with deep vein thrombosis, one patient with ischemic myocardium, one patient with pulmonary edema.

**Figure 7:**
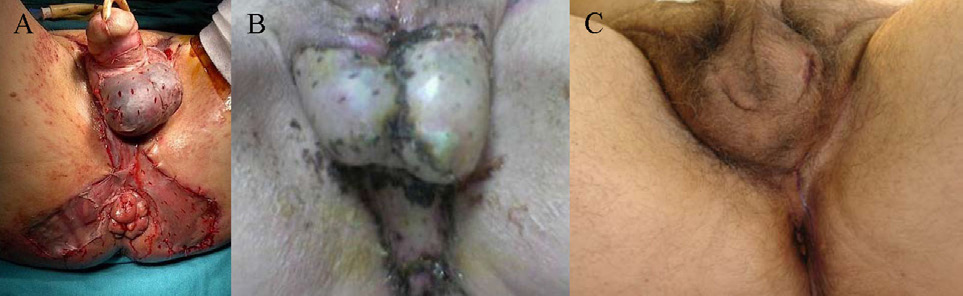
Skin graft reconstruction immediately after surgery **(A)**, long-term result after skin graft **(B)**, and wound healing by secondary intention **(C)**. The patients are in accordance with Figure 5.

From the 19 patients who survived wound healing was achieved by secondary intension in 14 patients (73.7%) and by reconstruction from plastic surgeons with a skin draft in 5 patients (26.3%) (Figure 7).

The median period of hospitalization, including the hospitalization in general surgery department, ICU, and plastic surgery department, was 27 days, 40 days in survivors, and 3 days in non-survivors, with the difference being statistically significant (*p* = 0.008). The difference in days of hospitalization is due to the fact that in non-survivors the death occurred early, during the first day of patient hospitalization. The median stay in the surgical department was 16 days, 25 for survivors and 3 for non-survivors. For the nine patients (37.5%) transferred following surgery to the ICU, the median ICU stay was 2 days while the median ICU for the all the patients was 0 days; however, some patients were in ICU for up to 27 days. For the patients submitted to skin graft, the median stay in the plastic surgery department was 24 days.

Regarding non-survivors all the patients died during hospitalization and earlier than 30 days hospitalization. So the in hospital, 30 and 90 days mortality rate was the same and was 20.83% (5 of 24 patients). The cause of death was severe sepsis and septic shock in three patients. One patient died from massive pulmonary embolism, and one patient died from generalized carcinomatosis, cancerous cachexia, and MODS.

Univariate analysis revealed as significant predictors of mortality, the presence of malignancy, low hematocrit, and low hemoglobin. However, in multivariate analysis, none of these was significant.

## Discussion

In 1883, a French dermatologist and venereologist, Jean Alfred Fournier, described a life threatening clinical condition characterized by progressive necrotizing fasciitis of the male genitourinary tract (1–14, 16–18, 26–36). This condition was officially named as FG despite the fact that it has been originally described by Baurienne in 1764, by Pouteau in 1783, by Jones in 1871, and later by Avicenna in 1877 (4, 6–17). According to Fournier, the disease is characterized by an abrupt onset of painful scrotal swelling and a rapid progression to gangrene in otherwise healthy men without any obvious or definite cause (3, 4, 7, 8, 10, 11, 13, 14, 17, 26, 32, 36). Many different terms including idiopathic gangrene of the scrotum, periurethral phlegmon, streptococcal scrotal gangrene, gangrenous erysipelas of the scrotum, and synergistic necrotizing cellulitis have been used to describe this clinical entity over the years (8, 9, 11–13). The term “necrotizing fasciitis” was first used by Wilson in 1952 for the description of inflammation of soft tissue occurring in superficial and deep fascia regardless of location (8). Initially, the term “Fournier gangrene” was used for the idiopathic gangrene of genitals of men; however, later this term began to be used for all almost the necrotic inflammations of the region (14, 16).

In the past, Fournier gangrene typically affected predominately male patients and developed rapidly, but nowadays it has been reported to be diagnosed in people of all ages, from newborn babies to the very elderly (4, 28). FG is a rare condition and affects around 1:7,500 (12, 33). While, Fournier’ gangrene is still considered by many a disease that affects only males, it is nowadays evident that females can be affected too and develop necrotizing fasciitis of the perineum and genitalia, although in many cases it was not termed FG, and that may be a reason why the disease in females is under recognized (8). However, most of the studies reveal a male predominance (male:female is 10:1) with a mean age of 50 years although high incidence of disease presentation in female patients with an increased percentage of 30% or even 47.7% have been reported (5, 11–13, 16, 17, 37, 38). Male predominance exists perhaps due to easier drainage of the female perineum via the vaginal route (17). While, disease predominance is affected by gender, mortality is not and males and females present with similar prognosis. However, despite the fact that the disease is less common in females, yet it is often more extensive, because of the female pelvic anatomic feature (39). In our series, there was an increased incidence of female patients (16.7%) possibly due to the fact that patients were treated by a general surgery department and not by a urology department. While the reported mortality rate of the disease was high between 30 and 50% (36), unfortunately even in our days and despite the development of modern medical therapies the mortality remains high reported in a range from 7 to 33% (32). In our series mortality was within the reported range in literature as it was 20.83%.

Fournier’s gangrene is an infection caused by aerobic and anaerobic bacteria, usually acting synergistically that spread along the subcutaneous and fascial planes across the perineum, scrotum and sometimes beyond these tissues leading to thrombosis of subcutaneous vasculature and skin necrosis (4, 7, 10). This typical clinical picture of gangrene generally starts in the scrotal region and rapidly spreads to the penis, perineum, and inner thighs (4). The disease most usually present with rapid onset followed by a fulminant progression, and rarely with an insidious onset followed by slow course (9). The local signs are those of inflammation including pain, heat, erythema and edema involving the scrotum in up to 93.3% the penis in 46.5% and the perineum and perianal region in 37.2% (40). However, in the present study, possibly due to the fact the data come from a general surgery department and not from a urology department, perineal and perianal involved was high (91.7 and 54.2%, respectively), and penis involvement was noted only in one patient (4.2%). Local inflammation signs were present in the majority of patients, but crepitus only in five of them (20.8%), which is accordance with the literature as crepitus can be found in 19–64% of cases (32, 37). Also, as the disease progresses, necrosis of the overlying tissue may become evident (9, 32, 40). Moreover, systemic signs, including fever, tachycardia and tachypnea, are usually present, and the diagnosis of SIRS can be made in up to 84.7% of cases (9, 18, 32, 40), as in our series were it was evident in 75% of cases, in whom the diagnosis of sepsis was established as all the patients were considered to have a clinically documented infection. In some cases, sepsis can progress to severe sepsis and even septic shock (37.5 and 12.5% in the current study, respectively). Laboratory studies usually reveal leukocytosis, thrombocytopenia, anemia, which are caused from sepsis (32). Anemia is a result of thrombosis, ecchymosis and decreased erythrocyte production leading to reduced functioning erythrocyte mass (32). In the present study, while the median hematocrit and hemoglobin was decreased, anemia was more evident in the non-survivors group and univariate analysis showed that it statistically significant affected mortality. Besides the complete blood cell count, biochemical studies should be performed including electrolytes, glucose levels, urea and creatinine, arterial blood gas to access pH, and coagulation studies (40). In various studies, variable laboratory data have been associated with mortality such as creatinine, calcium, lactate, and bicarbonate (40); however, in our series, possibly to the small sample, none of them differed statistically significant between survivors and non-survivors.

Imaging modalities can aid to confirm the diagnosis in ambiguous cases, reveal the underlying etiology and estimate the extent of the disease (13, 32, 40). Radiographs may demonstrate the presence of soft tissue air, even before it is evident in clinical examination (32, 40). It has been reported that it has a sensitivity of 90–100% in revealing soft tissue air in diabetics with necrotizing fasciitis, while clinical examination showed gas only in 19–64% of the patients (32, 40). However, in our case series gas in the soft tissue was present in 75% of the cases, which is in agreement with the fact that the absence of air in the soft tissue in the pelvic X-ray does not rule out the diagnosis of FG. In these cases, other imaging modalities including ultrasound, computed tomography, and magnetic resonance imaging can establish the diagnosis, by demonstrating the presence of air in the subcutaneous or soft tissue of the scrotum and the perineum along with soft tissue inflammation and thickening (9, 32, 40). Furthermore, these modalities can provide useful information in evaluating the extent of FG, and also they can reveal the underlying etiology of the disease (32). In the current study, one patient has been submitted to MRI before hospital admission, which confirmed the diagnosis by revealing the characteristic findings of FG, demonstrating that MRI, while due to extended time of the exam and increased cost is not an examination of choice, can establish the diagnosis of the disease.

In patients developing FG, there are frequently a number of comorbidities (1, 3–5, 7–18, 26, 29–36). Diabetes mellitus, alcoholism, immunosuppression, chemotherapy, chronic corticosteroid use, HIV, leukemia, cardiac disorders, systemic lupus erythematosus, obesity, liver disease, Crohn’s disease, and kidney failure are some of the comorbid risk factors for the development of FG (1, 3–5, 7–18, 26, 29–36). Diabetes mellitus is present in 20–70% of patients with Fournier gangrene (9, 11). Diabetes mellitus was also noted in increased frequency in our series (29.2%). However, despite the fact that various comorbidities have been associated with mortality (11, 18, 33), in the present study the only comorbid condition associated statistically significant with mortality was the presence of malignancy either solid or hematologic (*p* = 0.038). Also, to evaluate comorbid conditions in total and to strengthen their effect, the following clinical scores were calculated: CCI and Age-Adjusted CCI (AA-CCI) (41–43). CCI has been developed to predict mortality and measure 1-year mortality risk by assessing comorbidities and the burden of disease (41–43). The AA-CCI score, in which age is being considered an independent factor of mortality, can be generated by adding additional points for age (41–43). CCI and AA-CCI have been used as predictors of mortality in FG (18).

There are three routes that can be followed by the infection in FG (33). First, bacteria in the lower urinary tract can move to the paraurethral gland and corpus spongiosum and through the Buck’s fascia the infection can extend to Dartos, Colles’, and Scarpa fascia (7, 33). The infecting bacteria probably pass through Buck’s fascia of the penis and spread along the Dartos fascia of the scrotum and penis, Colles’ fascia of the perineum, and Scarpa’s fascia of the anterior abdominal wall (3, 7, 11, 26, 33). The second route for the infection begins around the rectum and spreads directly to the scrotum and testis through Colles’ fascia (3, 7, 33). As a third route, we can list the bacteria that are present on the skin and can penetrate the subcutaneous tissue with trauma (7, 33). The testicles are protected from the inflammation due to their unique vasculature from the testicular artery. So the suspected source of sepsis in Fournier gangrene can be idiopathic, colorectal, urogenital, dermatological, and of course traumatic (5, 8, 9, 11, 33). The commonest causes of FG are urogenital (urethral stricture, indwelling catheter, traumatic catheterization, prostatic biopsy, vasectomy, and perineal trauma), anorectal (perianal abscess, rectal biopsy, anal dilatation, hemorrhoidectomy, rectosigmoid malignancy, appendicitis, and diverticulitis), and gynecological (infected Bartholin’s gland, septic abortion, episiotomy wound, coital injury, genital mutilation) (5, 8, 9, 11, 33). Local trauma, extension of a urinary tract, and a perianal infection are the most common initial ports of entry (11). In the current study, the most common etiology was colorectal followed in frequency by cutaneous infections and abscesses and by urogenital. As in other series, so in ours etiology was not correlated with mortality (18).

All of the abovementioned factors in association with some bacterial species can easily be the start of a polymicrobial severe subcutaneous infection that begins adjacent to the portal of entry, which may be urethral, rectal or cutaneous (1, 3–18, 26, 29, 32–34, 44). Localized cellulitis progresses to a diffuse inflammatory reaction involving deep fascial planes. This bacterial infection results in microthrombosis of the small cutaneous and subcutaneous vessels leading to the development of gangrene of the overlying skin (1, 3, 5–9, 11–13, 16–18, 26, 29, 32–34, 44). Small subcutaneous vessels thrombosis and subsequent tissue necrosis have as a result low oxygen concentrations which lead to anaerobe growth (5). Moreover, these anaerobes and aerobes by acting synergistically produce multiple enzymes like heparinase, collagenase, hyaluronidase, streptodornase, and streptokinase, which cause tissue destruction (5, 9). The rate of fascial necrosis has been documented to be approximately as much as 2–3 cm/h (12, 32). This progression of tissue necrosis results from the endarteritis caused by the spread of the microorganisms (4, 7). These microorganisms in association with local edema, hypoxia caused by difficulty in local blood supply, which favors the development of anaerobic bacteria, produce hydrogen and nitrogen that accumulate in tissues causing crepitation and leading to a rapidly evolving necrotizing fasciitis of the scrotal soft tissue and of the perineum (4, 7). The responsible bacterial strains consist of both Gram-negative and -positive aerobic and anaerobic species, but anaerobes were identified less frequently: *E. coli, P. aeruginosa, Proteus, Klebsiella, Streptococcus* species, *S. aureus, Enterococcus, Clostridia, Bacteroides*, and less commonly MRSA and *Candida* in patients who were hospitalized for a longer time (1, 3–5, 7–13, 26, 29). In the current study, also more infections were polymicrobial, and the most commonly isolated bacteria were Gram negative, and specifically *E. coli*, then anaerobic bacteria, and finally Gram positive, but the type of bacteria did not affect mortality.

The cornerstones of therapy are the early recognition, rigorous hemodynamic support, aggressive resuscitation and intravenous hydration, urgent and aggressive surgical debridement of all necrotic areas, and antibiotics (12, 29, 45). The key to the treatment of Fournier gangrene is early and aggressive surgical debridement of the necrotic tissue, empirical broad-spectrum antibiotic therapy (third-generation cephalosporin, penicillin, and metronidazole) along with treatment of the predisposing conditions (13, 14, 26). Surgical debridement must aim in resecting all necrotic and infected tissue, and an aggressive approach should be adopted (14). While the time interval from disease onset to surgery, and especially the delay from hospital admission to surgery may be an important prognostic factor regarding mortality, not all studies have corroborated that (37, 46), as in the present series were there was statistical significant difference between survivors and non-survivors. Moreover, the extent of the disease, which is directly related to the extent of surgical debridement has also been correlated to mortality, once again not to be confirmed by other studies (46, 47) as in our series were the body surface involved by FG was not statistically significant related to mortality. Furthermore, regarding the number of surgical debridements performed in each patient, the data is still controversial as some studies found it to be a significant parameter of mortality, while others not (29, 46, 47), as we in our series.

Furthermore, protective colostomy, although still controversial, in some series has been considered important to achieve rapid healing because it eliminates fecal contamination (14). Recent studies have shown significantly lower mortality in the patient groups with protective colostomy compared with them without colostomy (15, 28, 29). This can easily be explained as the wound in the acute inflammatory phase can stay clean, and fecal diversion can prevent any further infection spread in the area (28). Moreover, the colostomy can affect the rapid improvement of nutritional status which is very important for containing inflammation, through the early enteral nutrition (15). A loop colostomy is always preferred instead of an intestinal stoma because of the formed and solid stools that are easily restricted, and there are fewer possibilities for contamination to the surrounding skin (15). However, it is claimed that even if destruction of the perirectal area occurs colostomy is never necessary (14). In our series we did not find any significant difference in survival in patients with and without colostomy. Moreover, in some cases a rectal diversion device can be used instead of colostomy to prevent fecal contamination of the wound (40).

In addition, the hyperbaric oxygenation helps marginally viable tissues survive. Its anti-inflammatory properties prevent ischemia, edema, reperfusion injury, and further complications of hypoxic tissues (13, 40). The debate regarding hyperbaric oxygen and mortality is still ongoing as there are data both showing reduced mortality and other showing no association between hyperbaric oxygen and mortality, as was the case in the current study (7). With the recent advent of the VAC system dressing, there seems to be a dramatic improvement with minimizing skin defects and speeding tissue healing (9). VAC is a wound care system based on the negative pressure vacuuming and has been used with great success to care many different types of wounds (2). VAC therapy involves the application of a sterile open-cell foam sponge to the wound, adding transparent adhesive drapes and a non-collapsible tube, which helps the connection to a portable pump that provides negative pressure to this air-tight environment of the wound (2, 11). VAC application promotes blood flow and creates a perfect environment for wound healing. VAC therapy reduces tissue edema and excess fluid, increases oxygen and defender wound cells (2). A negative pressure value of 125 mmHg was therefore selected for use in our study. In the current study mortality rates were not influenced by rectal diversion, hyperbaric oxygen treatment or VAC therapy. Regarding, urinary diversion, while urostomy may be suggested in some cases, satisfactory diversion can be achieved by urinary catheterization (13, 40) as in the current study.

The final goal in treating a patient with FG after the patient has recovered is the reconstruction of the extended defects caused by the aggressive surgical debridement, once granulation tissue has formed (45). A lot of methods have been described including healing by secondary intention, delayed primary closure, loose wound approximation, skin graft, and flaps (13, 30, 40). While secondary intention has been proposed for small wounds confined to the scrotum (13, 30, 40) and can lead to prolonged hospitalization, and deformity due to contracture we have successfully employed this method in our series in the management of more extended wounds. For larger defects either flap reconstruction or split thickness skin graft can be used (13, 30, 40). Skin graft is a simple procedure that can be done in one stage with a good cosmetic and functional result (13, 30, 40), as in the present series were this technique was employed in five patients by plastic surgeons.

For the prediction of mortality in FG that is to validated indexes the FGSI score and the Uludag FGSI. Also, for the same reason, the acute physiology, age, and chronic health evaluation II severity score (APACHE II) has been used along with the laboratory risk indicator for necrotizing fasciitis (LRINEC). The FGSI score, has been created in 1995 by Laor et al. by modifying the APACHE II score (26). There are nine parameters studied: heart rate, respiratory rate, temperature, hematocrit, leukocyte count, serum sodium, potassium, creatinine, and bicarbonate levels, and the deviation from normal is graded in a scale from 0 to 4 to a maximum of 36 points. The values are added to calculate the FGSI score (26). While its predictive value have been validated in a lot of series (18), and a cutoff point of 9 seems to provide a good discriminatory capacity regarding mortality, in our study due to the small sample, we did not find any statistically significant difference regarding the FGSI in survivors and non-survivors. The newer and novel Uludag FGSI, which was created by Yilmazar et al. in 2010, also takes into account age and disease dissemination to a maximum of 43 points (18). In contrast with FGSI, which accounts only for the patient’s acute physiologic status, APACHE II evaluates the patients both acute and chronic health status to predict mortality in severely ill patients with critical systemic disease (48). It has been used both for necrotizing fasciitis in general (48) and for FG specifically, in which a cutoff value of 20 has been proposed (45). Finally, the laboratory risk indicator for necrotizing fasciitis (LRINEC) that is based on the values of CRP, WBC, hemoglobin, serum sodium, serum creatinine, and plasma glucose has also been employed in an attempt to predict mortality for necrotizing fasciitis in any part of the body but also for the perineum (FG) (11, 49). A value of LRINEC <6 is related to low risk of mortality and ≥9 with a high risk, while a value in between with a moderate risk (49). In our case series, we could not find any statistically significant difference neither for APACHE II nor for LRINEC score between survivors and non-survivors. Possibly, our relatively low mortality rate can be in part attributed to the low median score of all the prognostic indicators, which were 5 for FGSI, 6 for UFGSI, 10 for APACHE II, and 5 for LRINEC.

Fournier’s gangrene represents a severe disease with increased morbidity and mortality. Morbidity rates as high as 80% have been reported with mean hospital stay of 31–35 days (10). The median hospital stay in our in our series was 27 for all patients, but 40 for survivors only. Regarding morbidity, there is still an ongoing debate about the incidence of incontinence following aggressive surgery. Aggressive surgery is mainly related to increased rate of secondary wound infection and the need for skin grafts to achieve wound healing (11). Fecal incontinence is not a commonly reported complication of aggressive debridement as muscle involvement is rarely present (7). Very few studies report long-term outcomes in patients with FG, and most of them focus on chronic pain, disfigurement and sexual dysfunction leading also to psychosocial problems of these patients (9, 50). While fecal incontinence may present simultaneously with the disease onset in patients with an extensively damaged anal sphincter in up to 40% who would require a stoma, following stoma closure no morbidity was recorded (38). Fecal incontinence seems to be temporary and may affect some of the patients (9).

In conclusion, FG remains a surgical and urological emergency with increased morbidity and mortality even nowadays and the general surgeon must be familiar with it, as its early recognition and aggressive surgical treatment, along with antibiotic therapy and rigorous resuscitation, are the basis of this severe condition’s successful management.

## Ethics Statement

Due to the retrospective nature of the study and as there was no research intervention, the current study was exempt from this requirement.

## Author Contributions

OI: study design, study supervision, data collection, analysis, interpretation, introduction, methodology, results, and discussion sections, and critical revision for important intellectual content. LK: data collection, introduction, methodology, results, and discussion sections. DT: data collection, analysis, interpretation, methodology and results sections. IS, AC, AG, and IV: data collection, analysis, interpretation, methods and results sections. SS, NS, and SP: data collection, methods and results sections. GP, MP, EK, and IM: study conception and design, data collection, methodology, results, and discussion sections. KT: study conception and design, study supervision, introduction, methodology, results, and discussion sections, and critical revision for important intellectual content.

## Conflict of Interest Statement

The authors declare that the research was conducted in the absence of any commercial or financial relationships that could be construed as a potential conflict of interest.
